# Combined quadriceps tendon reconstruction and total knee replacement with computer navigation: a case report

**DOI:** 10.1186/s13256-022-03265-2

**Published:** 2022-02-03

**Authors:** Imran Haruna Abdulkareem, Perry Liu, Ajeya Adhikhari, Deiary Kader

**Affiliations:** 1 Southwest London Elective Orthopaedic Centre (SWLEOC), Epsom Surrey, UK; 2 Academic Unit, Southwest London Elective Orthopaedic Centre (SWLEOC), Epsom Surrey, UK

**Keywords:** Quadriceps, Patellar, Extensor mechanism, Tendon, Rupture, Repair, Reconstruction, Osteoarthritis (OA), Total knee replacement (TKR), Computer navigation, Robotics, Outcomes

## Abstract

**Background:**

Chronic quadriceps tendon rupture is technically challenging for surgeons due to scarring and tendon retraction. The presence of concurrent ipsilateral knee osteoarthritis compounds the issue even further. Although a combined presentation is rare, treatment options to manage each coexisting pathology simultaneously are scarcely reported. We describe the case of a 67-year-old Caucasian male who had such a presentation, and was subsequently treated with a one-stage extensor mechanism autograft reconstruction and total knee replacement with computer navigation.

**Case:**

The patient was a 67-year-old male Caucasian, who had previously sustained an acute rupture of his right quadriceps tendon that was adequately repaired 6 months prior. Despite an initial satisfactory result, he reported deterioration in his mobility in the few months thereafter, with worsening knee pain. His comorbidities consisted of hypertension, asthma, and a body mass index of 40.4 kg/m^2^. Otherwise, there were no risk factors for tendon rerupture. Clinical examination later revealed a large palpable gap in the right suprapatellar region and weakness of active knee extension. No traumatic cause for this new presentation was identified. Suspicion of a chronic quadriceps tendon rupture was confirmed on radiological imaging, but the investigations also noted the presence of severe tricompartmental osteoarthritis of the ipsilateral, native knee joint. The combined procedure took place in one surgical sitting. The total knee replacement with patella resurfacing was performed first and assisted by computer navigation. The quadriceps tendon reconstruction was then conducted sequentially using the patient’s hamstring tendons (semitendinosus and gracilis). The tensile strength was reinforced with use of a Ligament Augmentation and Reconstruction System (LARS) ligament. Initial outcomes were excellent, and these results were maintained at 6 months postoperatively, with the patient reporting no pain and having full range of movement.

**Conclusion:**

Our techniques used have not previously been reported, but are successful options in treating coexisting chronic quadriceps tendon rupture and ipsilateral knee osteoarthritis. The advantage of using computer navigation with an extramedullary femoral jig can lead to improved accuracy of bone cuts, which is important in the presence of anatomical disruption. Chronic failures of the extensor mechanism require different approaches depending on the inherent and underlying pathology. We feel that the multidisciplinary team approach to the management and use of two surgeons with differing expertise added to the successful outcome of this complex case.

## Introduction

The incidence of isolated quadriceps tendon rupture is low and is associated with certain risk factors such as smoking, steroid use, diabetes mellitus, inflammatory conditions, connective tissue disorders, and sporting overuse [1]. Such injury is functionally debilitating for the patient, surgically challenging, and associated with poor outcomes. In general, tendon pathologies present as a spectrum [1], and the treatment options range from conservative management for tendinosis and partial rupture, to surgical intervention for acute, complete ruptures as well as delayed presentations, including chronic and neglected cases [1].

Chronic or neglected quadriceps tendon rupture is often a clinical conundrum for surgeons. The technical challenges posed by the nature of the lesion, scarring, and tendon retraction necessitate complex surgical procedures and augmentation techniques for reconstruction [2]. The difficulty is compounded by the presence of osteoarthritis (OA) in the knee joint, and in such instances, it may be beneficial to perform both a quadriceps tendon reconstruction and total knee replacement (TKR) simultaneously [3].

However, although a combined presentation is uncommon, the management of chronic quadriceps tendon rupture and concurrent knee OA is poorly documented in the orthopedic literature. Most relevant studies only comment on tendon rupture as a complication after TKR surgery for OA, rather than as a coexisting pathology preoperatively. For example, Dobbs *et al.* [4] reported an incidence rate of 0.1% (*n* = 24) for quadriceps tendon rupture postoperatively in a cohort of 23,800 primary TKR patients. They concluded that such injury is an uncommon sequel [4] to primary knee arthroplasty, but the risk of complications was high following occurrence.

We present a rare case of a middle-aged Caucasian gentleman with chronic rupture of his right quadriceps tendon that was complicated by concurrent severe right knee OA. At the time of submission, a literature search revealed only one other case [9] describing the same presentation, but the treatment methods of Piatek *et al.* differed greatly to ours. Our patient underwent a combined tendon reconstruction (with hamstring autograft) and TKR (assisted with computer navigation) in one sitting. This case report will discuss the procedural aspects and challenges of having to perform a dual procedure in this way. The techniques used have not previously been reported. Given the paucity of evidence describing similar cases, this report adds to current orthopedic literature and presents a unique way for how surgeons may choose to proceed with this complex and challenging presentation.

## Case report

### Background

The patient was a 67-year-old Caucasian gentleman, who was assigned an American Society of Anesthesiologists (ASA) grade of 3 (based on his obesity, hypertension, and asthma). He was classified as severely obese in accordance with his body mass index (BMI) of 40.4 kg/m^2^ (height 173 cm, weight 121 kg).

Aside from these comorbidities, there were otherwise no risk factors for tendon rupture or clinical evidence of inflammatory conditions that could have increased his overall risk. He initially had an acute, nontraumatic rupture of his right quadriceps tendon without any precipitating cause, and the tendon was repaired in the private sector in November 2019 after a delayed presentation of more than 6 weeks.

At the time of the initial surgery, there were several observations of note:

1. The patellofemoral joint (PFJ) showed evidence of grade IV osteoarthritis.
2. The rupture was seen to have extended into the vastus lateralis and lateral retinaculum.
3. The torn ends of the tendon were degenerate and of poor quality.

Subsequently, the injured ends of the quadriceps tendon were freshened, debrided, and repaired with a modified Kessler’s technique (using an Arthrex fibertape) and reinforced with a continuous vicryl end-to-end suture. The medial parapatellar retinaculum was also repaired.

The intraoperative result was satisfactory, and flexion of the knee joint up to 90° was possible. He was discharged with a hinged knee brace, which was locked in extension for the first 4 weeks postoperatively, with a plan to gradually increase his range of motion (ROM) thereafter. Following an intense course of physiotherapy and rehabilitation, a good outcome was achieved at first. According to the patient, he returned to full unaided mobilization and was able to flex and extend his knee without any difficulty (clinical records of this were not available to us as the patient was initially treated elsewhere).

### Clinical presentation

In December 2020 (over 6 months following discharge), he was referred to our care by his general practitioner after presenting to them with signs and symptoms that were consistent with a chronic rupture of his quadriceps tendon. No traumatic cause for this presentation was identified. Symptomatically, he now reported moderate discomfort in his right knee as well, but was pain free on the contralateral side and in his hip joints. Despite this, his mobility was impaired to the extent that he relied on a walking stick to assist with his activities of daily living (ADLs). He was clinically well otherwise, with no history of fever or infection either locally (around the surgical site) or systemically.

Clinical examination demonstrated an unsteady gait. On closer inspection, there was a mature, well-healed surgical scar over his right knee, with no evidence of infection or inflammation. There was a large palpable gap in the suprapatellar area but minimal swelling and effusion in the knee joint itself. Wasting of the ipsilateral quadriceps muscle was also noted. He was found to have weakness of active knee extension and was unable to straight leg raise (SLR). However, he had a reasonable flexion range from 0° to 100°, which was mostly asymptomatic. Further examination was unremarkable.

**Fig. 1:**
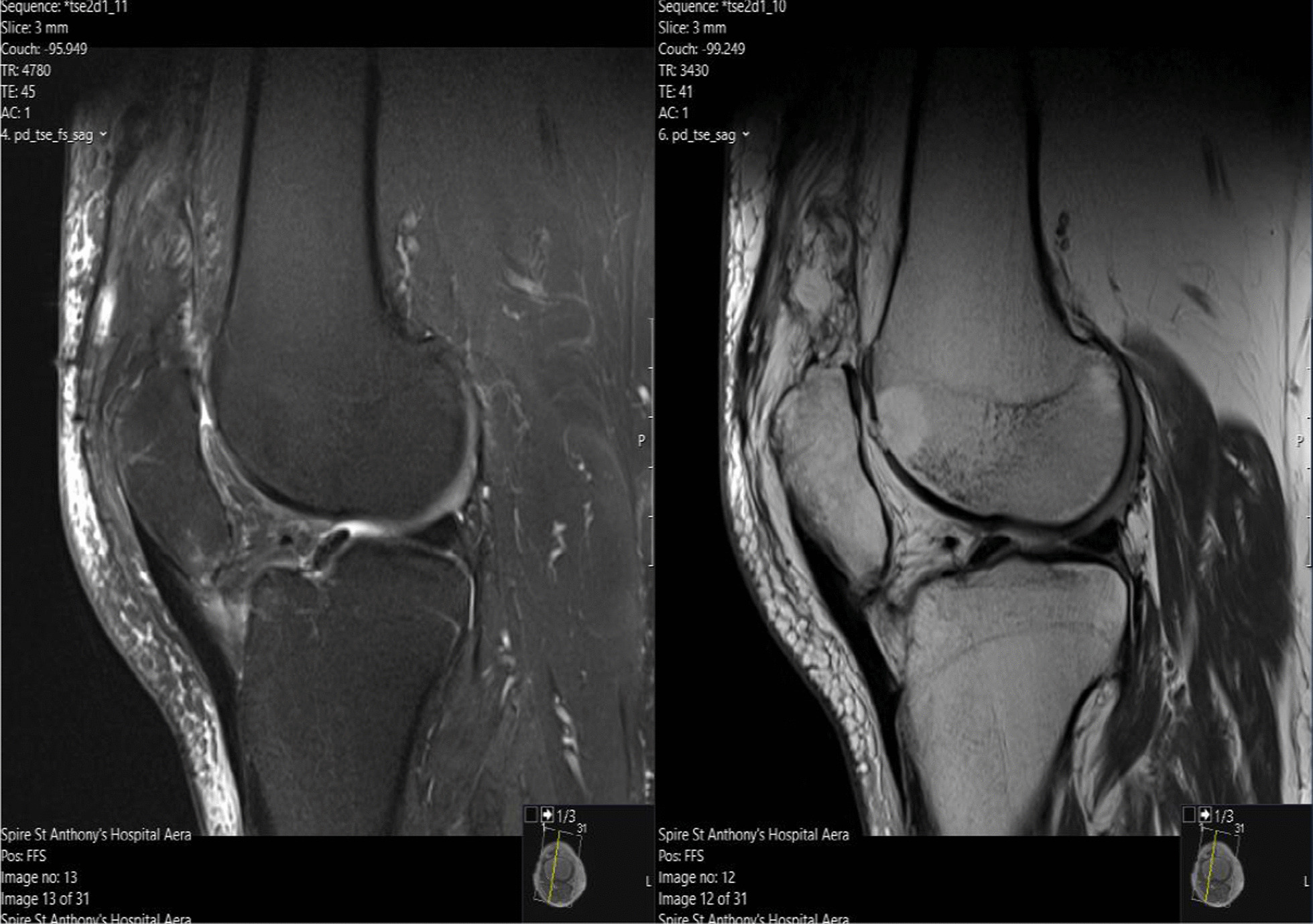
T1 and T2-weighted Magnetic Resonance Imaging  showing chronic right knee quadriceps tendon rupture, with tendon retraction, scarring, and fluid signal

**Fig. 2:**
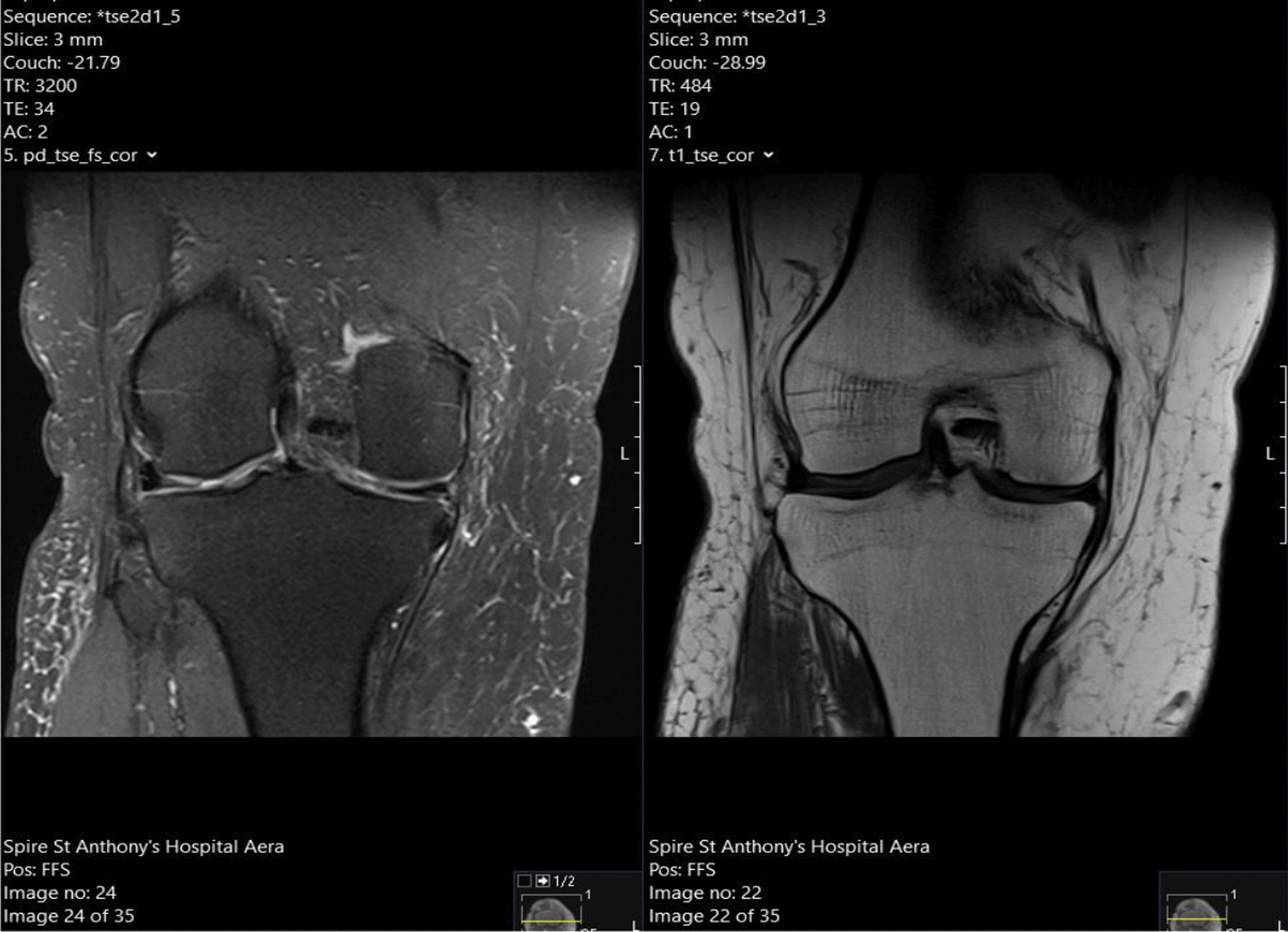
T1 and T2 Magnetic Resonance Imaging scan showing thinning and fragmentation of the articular surface in keeping with osteoarthritis

Considering these findings, the patient proceeded to have a magnetic resonance imaging (MRI) scan of his right knee, which confirmed chronic rupture of his quadriceps tendon. It showed that the ruptured tendon had retracted proximally, leaving a big residual space in the suprapatellar region with patella baja. Significant scarring and fluid signal were also seen within the rupture gap, which were radiographic signs consistent with the diagnosis (Fig. 1). Of significance, the imaging also revealed severe progressive tricompartmental OA of his right knee, associated with the chronic quadriceps tendon rupture at the distal musculotendinous junction above the patella (Fig. 2). A complete set of preoperative blood tests including inflammatory markers (C-reactive protein and erythrocyte sedimentation rate) were performed, all of which were within normal limits.

Following the results of these investigations, the patient was reviewed again in May 2021. An open discussion was held to ascertain their preferences regarding the surgical management of their dual problem of chronic quadriceps tendon rupture and severe knee OA. At this point, it was noted that his knee pain had deteriorated further as well.

In the meantime, a multidisciplinary team (MDT) discussion had been conducted in our unit to determine how best to proceed. We considered whether it was best to reconstruct the tendon first and perform the TKR at a later stage, or to perform both procedures in one sitting. Due to the severity of the OA and chronicity of the tendon rupture with subsequent retraction and scarring, the expert consensus was in favor of performing the two procedures in one sitting. This would thereby improve the patient’s chances of a good outcome. Furthermore, if only an isolated tendon repair was to be performed, a TKR would not be feasible for a prolonged period thereafter to allow for the full extensor mechanism to recover sufficiently. Considering the patient’s worsening symptoms, the dual procedure was deemed to be in their best interests.

The patient agreed with the proposed plan and was counseled accordingly. The expectations of surgery (including the possibility of permanent stiffness with limited knee flexion), intraoperative plan, and rehabilitation goals were discussed. The patient was also made aware that if the operation were to be unsuccessful, the only remaining options would be conservative management or a knee arthrodesis. Willing to accept the risks presented to him, the patient gave his informed consent to proceed with a right quadriceps tendon reconstruction and primary TKR in one sitting.

## Surgical procedure and intraoperative findings

**Fig. 3:**
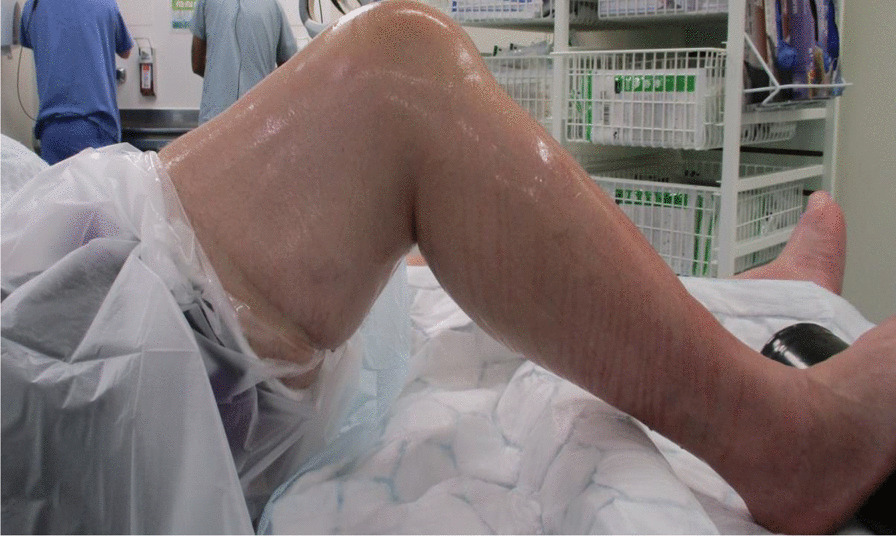
Intraoperative set-up for the patient about to undergo total knee replacement

After preoperative assessment and counseling, the planned procedure took place. A spinal block with light sedation was the anesthetic of choice. The patient was positioned supine on the operating table with the appropriate foot and lateral supports for optimal surgical access (Fig. 3). A high tourniquet was applied over the thigh to minimize the risk of bleeding. This was followed by routine cleaning and draping of the whole leg.

**Fig. 4:**
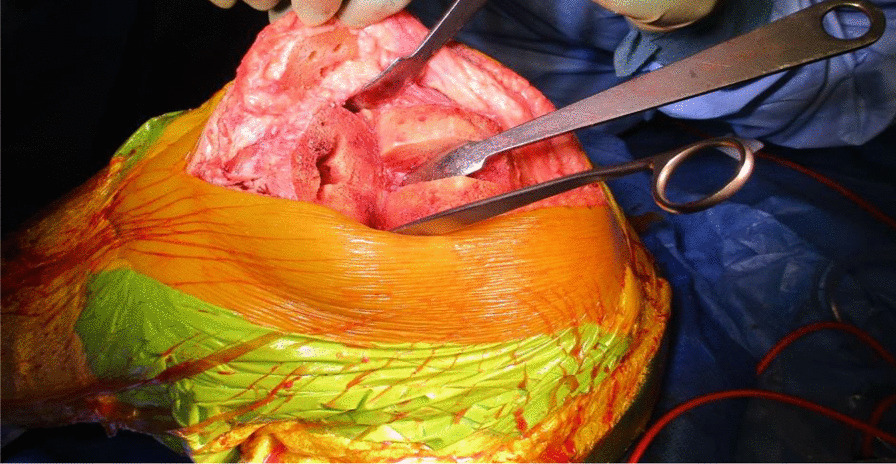
Intraoperative picture showing the completed femoral, tibial, and patella preparation, performed with the aid of Knee 3 computer navigation

**Fig. 5:**
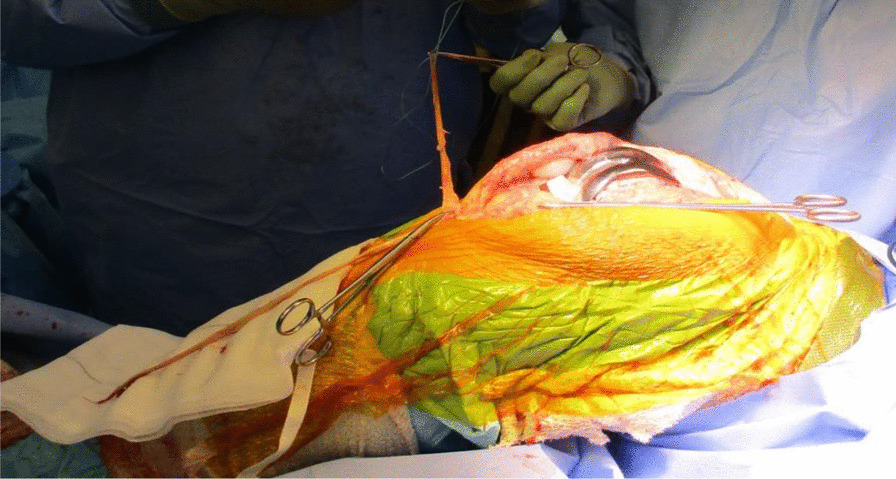
Intraoperative picture showing the completed total knee replacement and ipsilateral autograft hamstring harvest

The TKR was performed initially by one of the senior authors (AA), through a standard medial parapatellar approach over the healed surgical scar and using the Knee 3 Brainlab computer navigation, with a pin-less femoral extramedullary jig for assistance. The DePuy PFC Sigma CR Knee was the favored prosthesis, and the patella was also resurfaced. The wound was irrigated meticulously, and fresh drapes were reapplied to minimize the risk of wound contamination (Figs. 4 and 5).

**Fig. 6:**
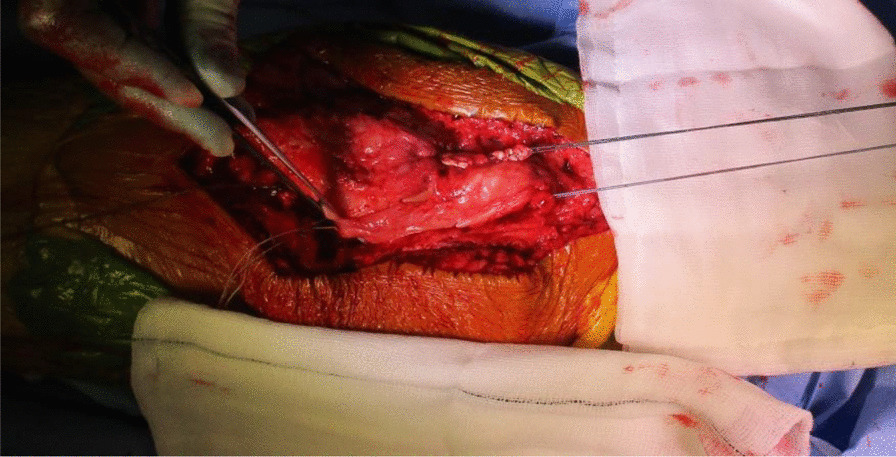
Showing hamstring tendons whipstitched and secured to the proximal patella with suture anchors

**Fig. 7:**
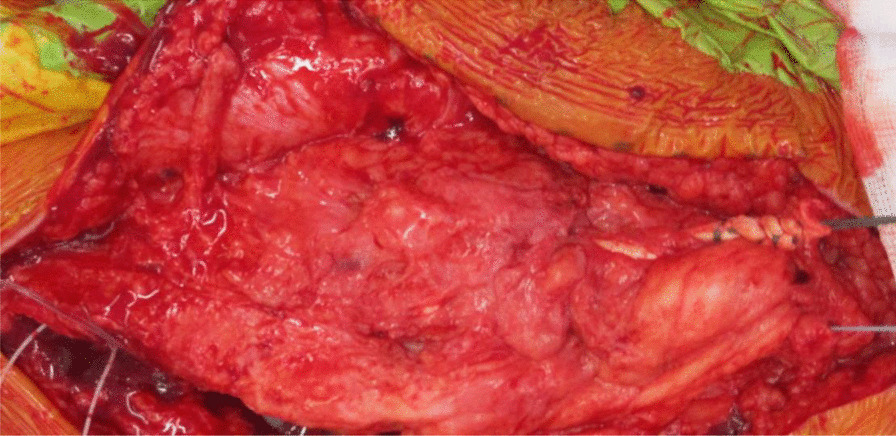
Hamstring tendons have been used to reconstruct the quadriceps tendon

**Fig. 8:**
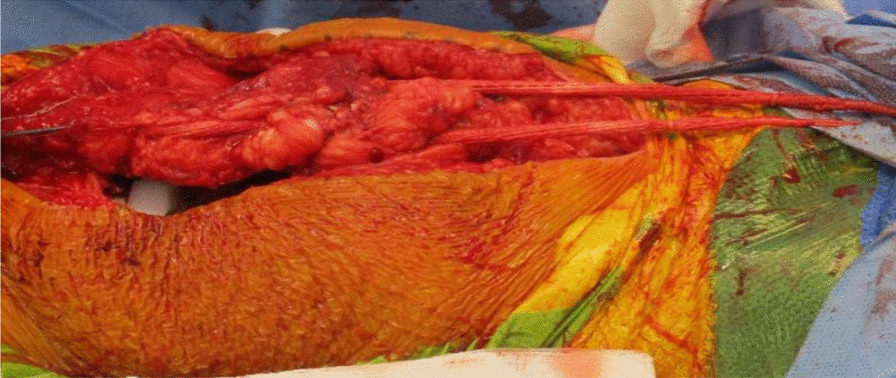
Ligament Augmentation and Reconstruction System (LARS) ligament used to reinforce and strengthen the quadriceps tendon reconstruction

**Fig. 9:**
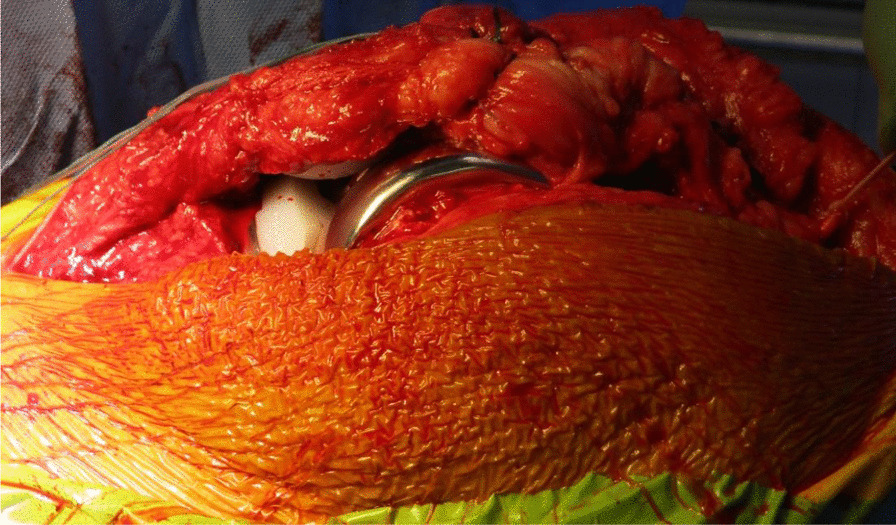
Shows the final quadriceps tendon reconstruction and total knee replacement, before wound closure

The quadriceps tendon reconstruction was then conducted sequentially by the second senior author (DK). The torn ends of the tendon were initially mobilized and released. Scar tissue was debrided to help improve the position and tracking of the patella after the reconstruction. The hamstring tendons (semitendinosus and gracilis) were harvested through the same surgical incision used for the TKR (Fig. 5). The two tendons were cleaned, whipstitched together, and attached to the proximal pole of the patella by G2 suture anchors. The hamstring graft was then weaved and sutured into the quadriceps tendon in a figure of eight configuration (Figs. 6 and 7). The Ligament Augmentation and Reconstruction System (LARS) ligament was also used to reinforce the reconstruction by passing it distal to the lower pole of the patella intrasubstance, and on the ventral side of the tendon, to improve the overall tensile strength (Figs. 8 and 9).

The knee ROM intraoperatively was 0–100°, with minimal tension over the reconstructed tendon, although there was still some residual patella baja observed. The retinaculum and rest of the wound were closed in layers after liberal washout with normal saline. The patient also received two doses of antibiotics as per local guidelines.

The knee was then placed in a ROM brace, which was to be locked in extension to allow for full weight-bearing for 4 weeks but able to flex up to 50° passively while sitting or engaging with physiotherapy during that timeframe. He was also advised to avoid any straight leg raise activity during the same period to protect the extensor mechanism. After an uncomplicated postoperative stay of a few days, the patient was discharged home.

The clinical assessments at various follow-up intervals are summarized as follows:

Day 10 postoperatively

**Fig. 10:**
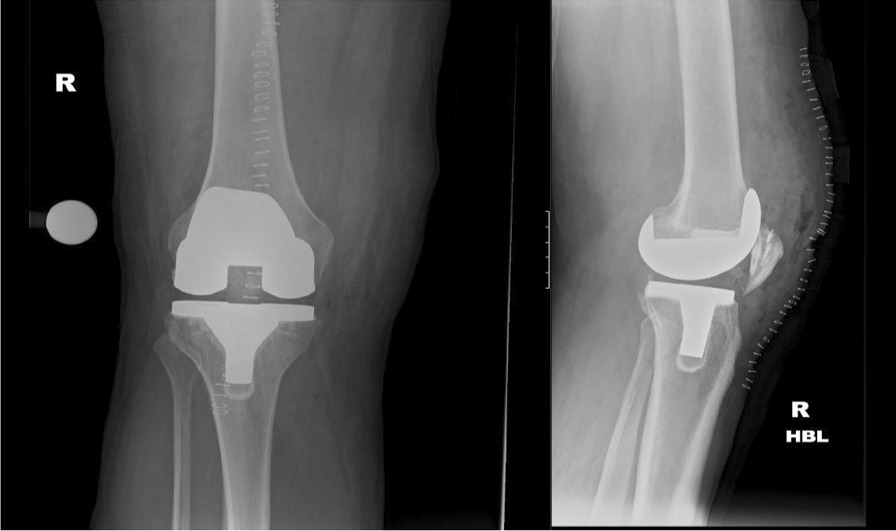
Showing immediate postoperative radiographs, which confirm adequate prosthetic implantation and positioning

- Skin clips were removed, and the condition of the wound was satisfactory.
- He had some residual postoperative swelling and pain with a visual analogue scale (VAS) of 2/10.
- Initial postoperative x-rays showed good position and implantation of the prostheses (Fig. 10). There was still some residual patella baja due to the chronicity of the quadriceps tendon rupture and retraction of the tendon ends. However, this was deemed acceptable given the minimal functional limitation.
- The patient was advised to continue physiotherapy and weight reduction measures.

Six weeks postoperatively

- The wound was completely healed with no signs of infection.
- He was completely pain free with VAS of 0/10.
- He achieved a flexion of 60° and an SLR of 0–30°. (He had been allowed to start gentle active knee extension exercises after 4 weeks).
- The brace was further unlocked to allow for full flexion, and he was advised to continue physiotherapy.

Three months postoperatively

**Fig. 11:**
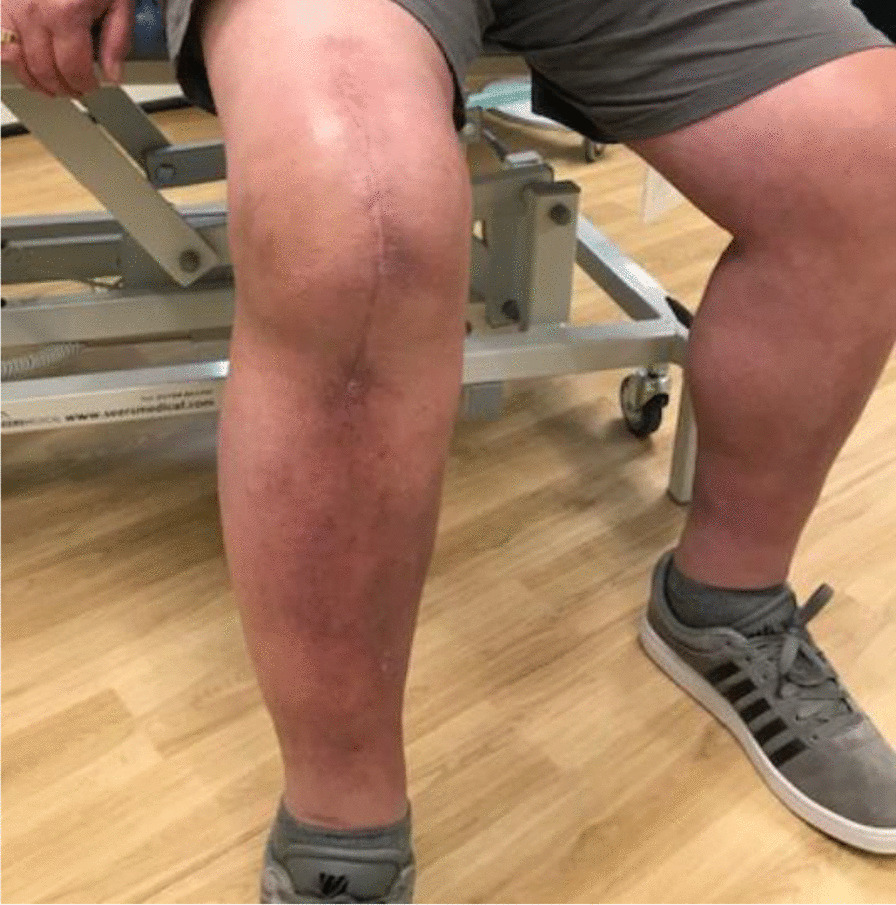
Showing a well-healed surgical scar and good knee flexion at 3 months postoperatively

**Fig. 12:**
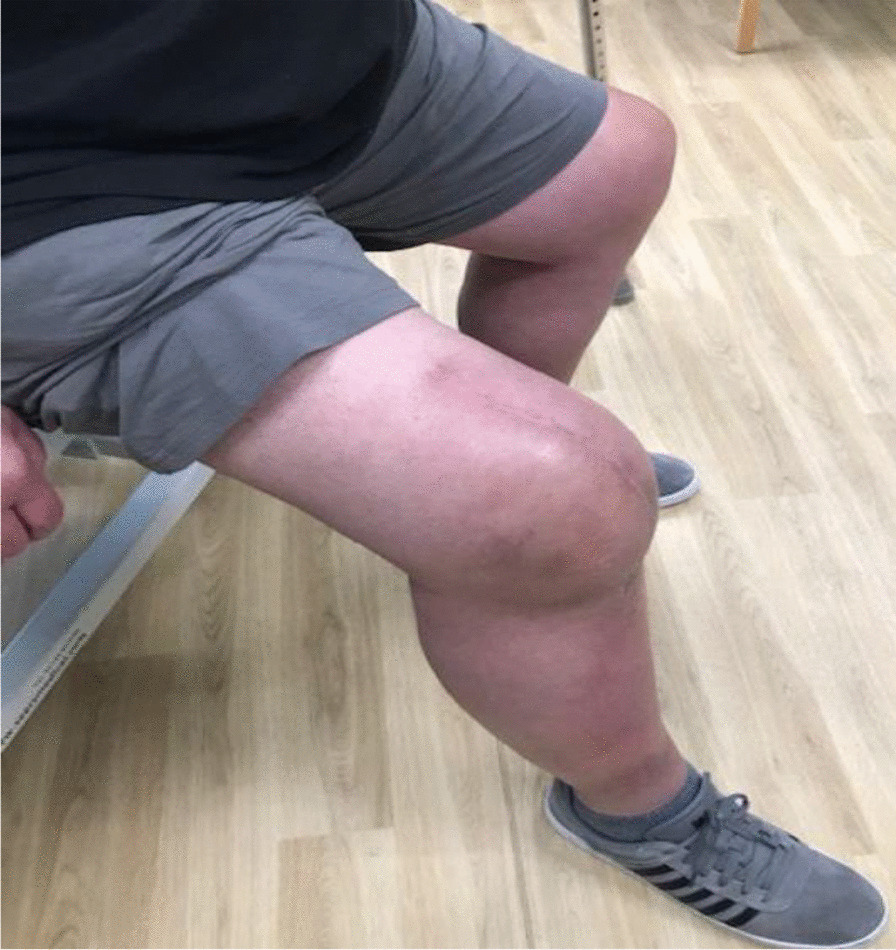
Side view showing the patient able to flex his knee to 90° at 3 months postoperatively

**Fig. 13:**
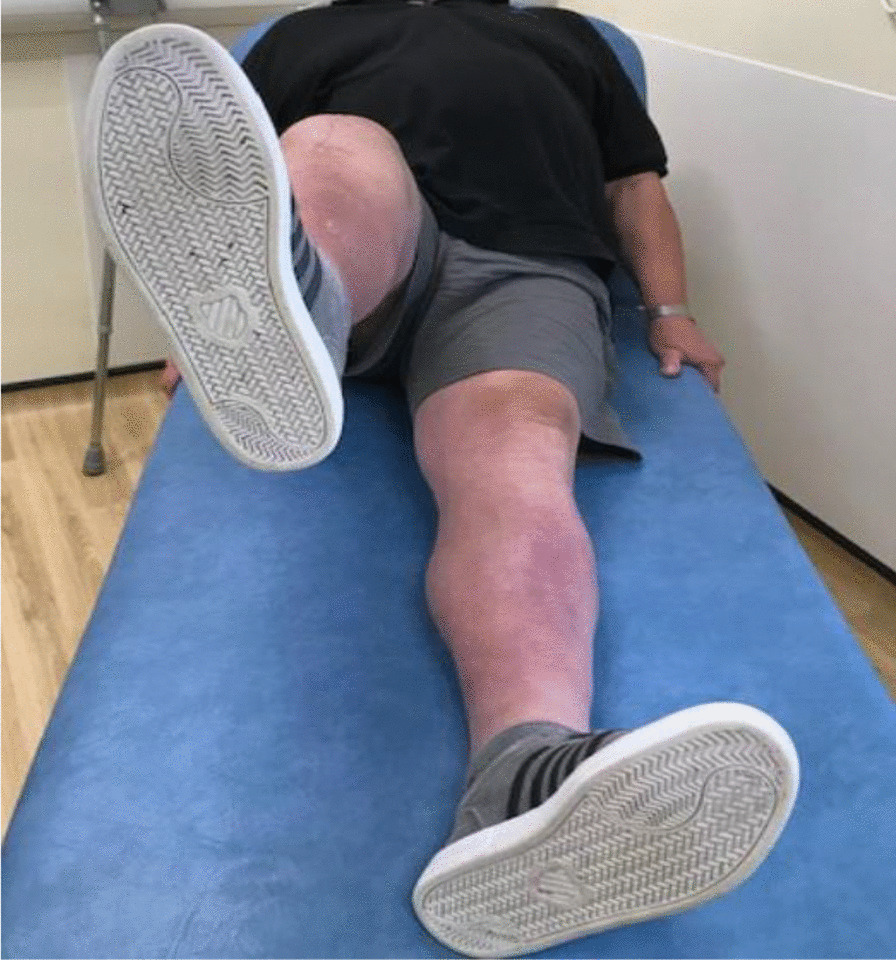
The patient demonstrating an straight leg raise of up to 45° at 3 months following the quadriceps tendon reconstruction

**Fig. 14:**
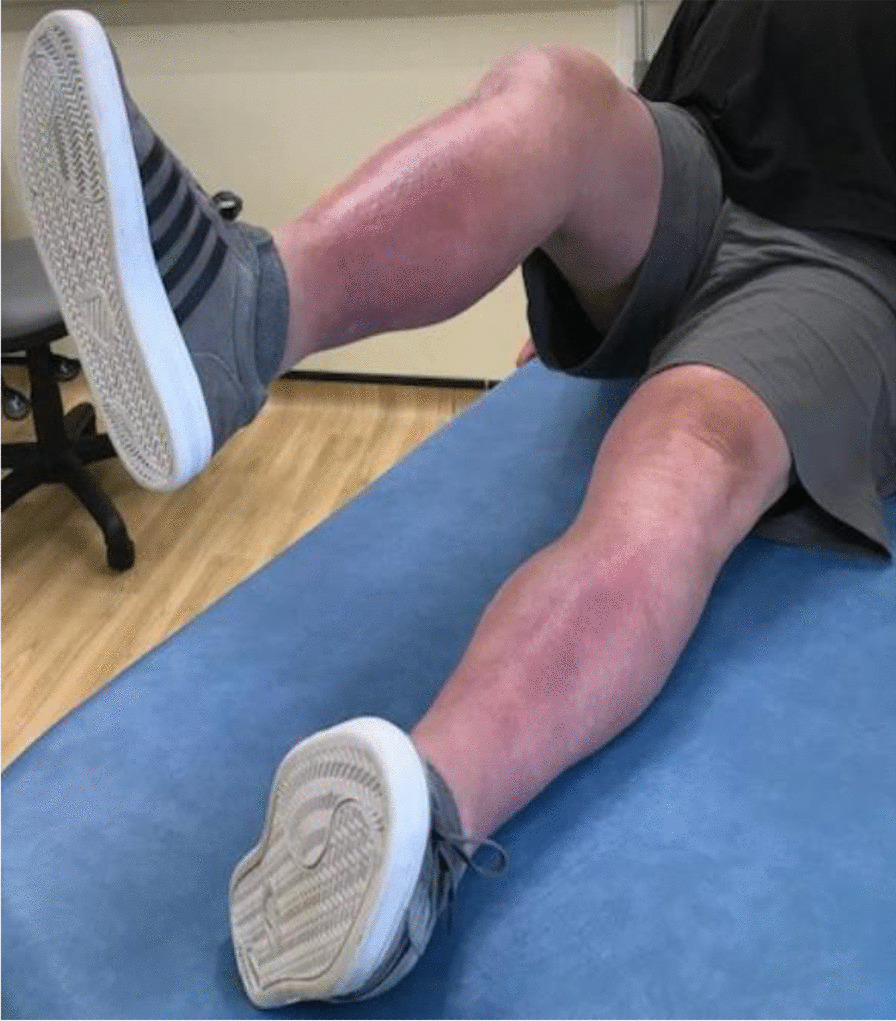
Side view showing straight leg raise and residual fixed flexion deformity at 3 months after the surgery

- The wound was completely healed.
- The patient was able to walk unaided without any symptoms or difficulty.
- There was active knee flexion of up to 90° (without the ROM brace), an SLR of 45°, and an extension lag of less than 10° (Figs. 11, 12, 13 and 14).
- The patient was very pleased with his clinical results, and he was allowed to resume activities as per his premorbid status.

Six months postoperatively

- Absence of pain (VAS 0/10) was maintained.
- He had continued with full unaided mobilization.
- He demonstrated flexion of at least 100°.
- The extension lag had marginally increased to 10–15°. Some weakness of his quadriceps muscle was noted, and he was given exercises to strengthen his extensor compartment accordingly.
- The patient remained pleased with his surgery and did not feel limited with his ADLs.

He will continue to be followed up for a minimum of 2 years, and his recovery is expected to remain uncomplicated.

## Literature review and discussion

We present a case of chronic quadriceps tendon rupture in a middle-aged Caucasian patient with ipsilateral knee osteoarthritis (OA), which were both managed at the same time with quadriceps tendon reconstruction and computer-navigated total knee replacement (TKR). Although a combined presentation is rare, information regarding the treatment of coexistent quadriceps tendon rupture and ipsilateral knee OA is very scarce in the orthopedic literature, with most publications focusing on extensor mechanism disruption following TKR [4]. Although similar cases about extensor mechanism injury with coexistent OA have been described, our specific techniques have not previously been published. Our report adds value by presenting an efficacious way of how to treat these difficult coexistent pathologies.

Only Piatek *et al.* [9] have reported a case like ours with their management of a 51-year-old male patient with knee OA, complicated by chronic quadriceps tendon rupture. In this article, the patient was treated with simultaneous conventional TKR and extensor mechanism allograft reconstruction, all fresh-frozen and nonirradiated. At final follow-up, he had painless ROM and was able to maintain active knee extension [9]. Although the presentation of this case is the same as ours, the treatment methods for both the tendon rupture and knee OA are different.

A relevant but unidentical case was presented by Zhang *et al.* [2], who described injury to a different part of the knee extensor mechanism (chronic patella tendon rupture) and ipsilateral knee OA. The 67-year-old man underwent conventional TKR and V–Y quadricepsplasty with Krackow suture technique at the same time. At 10 month follow-up, there were vast improvements in both the American Knee Society (AKS) and Hospital for Special Surgery (HSS) knee scores. This case differs from ours because their patient had patella tendon rupture rather than quadriceps tendon rupture, and again, the treatment methods for both the tendon rupture and knee OA were different from our methods.

On the other hand, there is a growing body of literature about extensor mechanism disruption following TKR surgery, as highlighted by Vyas *et al.* [5] in a recent review article. They identified that previous knee arthroplasty or knee arthrotomy, multiple steroid injections, patellar malpositioning, and lateral retinacular release at the time of the TKR were local risk factors for tendon rupture. Systemic precipitants included comorbidities such as rheumatoid arthritis, diabetes mellitus, chronic renal failure, obesity, and hyperthyroidism [5].

Other authors also described various treatment options available for such injuries, including allografts [6]^,^ (for example Achilles’ tendon [7]) with or without bone, and synthetic meshes [1]. Many studies have also shown good results with the use of allograft, such as the case series by Burnett *et al.* [8], on extensor mechanism injuries post TKR. However, despite the evidence base on this topic, articles describing the management of chronic patella or quadriceps tendon rupture with coexisting OA (as in our case) are scarce. In particular, the intraoperative strategies used to correct both defects in the same sitting are poorly documented.

Like Piatek’s case, we also adopted a combined surgery as it was advantageous to perform both procedures simultaneously. Only a single anesthetic and hospital admission were required, which reduced unnecessary exposure for the patient and the risk of additional hospital-acquired complications or morbidity during the recovery period. This strategy is particularly important given the ever-changing climate amidst the current global COVID-19 pandemic. A single admission is more convenient for patients as well, by allowing them to fulfill any working commitments or social activities without the need to take additional time off from work and family.

Unlike Piatek’s case [9], however, our intraoperative strategies differed in several ways. The process was facilitated by two senior surgeons which was favorable for the patient as it increased the efficiency of each procedure independently. From a technical point of view, further safety and accuracy of implant positioning were achieved by performing the TKR with the aid of computer navigation (Smith and Nephew Knee 3 Brainlab navigation system). The software was able to assist the operator by providing real-time feedback and thereby improved the accuracy of bone cuts, implant sizing, and positioning. A pin-less femoral extramedullary jig (Smith and Nephew) was also utilized, as it is known to be particularly useful in the presence of severe femoral or knee deformities and can minimize the risk of fat embolism compared with intramedullary femoral jigs [10]. These authors also mentioned that when computer navigation is used in TKR, 85% of the implanted knee replacements had ideal or very good alignment, compared with 55% in traditional TKRs without computer navigation [10]. A recent systematic review and meta-analysis also revealed that computer navigation-assisted knee replacement had improved outcomes when compared with conventional knee replacement, at mid-term follow-up [11].

With regards to the tendon reconstruction itself, we chose to perform this with autograft instead of allograft by harvesting the ipsilateral hamstring tendons (semitendinosus and gracilis). This was then strengthened with the synthetic Ligament Augmentation and Reconstruction System (LARS) ligament, as we felt that this would help to promote earlier mobilization postoperatively. The technique was found to be very secure and allowed knee flexion of up to 100° during the procedure. The autograft was taken through the same knee incision and so there was minimal donor site morbidity. Although there is clinical evidence to support the use of drill holes for tunneling and securing the autograft through the patella [12], we were not able to perform this because of the presence of patella resurfacing and cement mantle from the TKR. This presented a technical challenge of carrying out a TKR (with patella resurfacing) and quadriceps tendon reconstruction in one sitting. Despite this, the use of suture anchors and Ligament Augmentation and Reconstruction System (LARS) ligament augmentation as an alternative solution yielded a satisfactory outcome for our patient at 3-months follow-up and thereafter (Figs. 11, 12, 13 and 14).

## Conclusion

To the best of our knowledge, the intraoperative techniques described in this case are novel ways for managing coexisting chronic quadriceps tendon rupture and ipsilateral knee OA. With the ever-increasing technological advances in orthopedic surgery, we hope that our report is of educational value to the readers and something that may be considered in similar case presentations.

The techniques that we adopted had several advantages. The use of computer navigation with an extramedullary femoral jig led to improved accuracy of bone cuts, which was important in the presence of anatomical disruption. The hamstring tendon autograft was also easily accessible through the same surgical incision as the TKR, which allowed for the combined surgery to take place simultaneously.

Such techniques should be easy to teach and reproduce. Moreover, we were able to demonstrate efficacy with a good functional outcome and patient satisfaction at 6-months follow-up. On the other hand, more information will be needed on this topic to help guide best practice moving forwards, when faced with these two challenging and complex problems at the same time.

## Data Availability

All relevant images and clinical photographs have been included as a separate file in the submission.
